# Balancing costs and benefits at different stages of medical innovation: a systematic review of Multi-criteria decision analysis (MCDA)

**DOI:** 10.1186/s12913-015-0930-0

**Published:** 2015-07-09

**Authors:** Philip Wahlster, Mireille Goetghebeur, Christine Kriza, Charlotte Niederländer, Peter Kolominsky-Rabas, on behalf of the National Leading-Edge Cluster Medical Technologies ‘Medical Valley EMN’

**Affiliations:** Interdisciplinary Centre for Health Technology Assessment (HTA) and Public Health (IZPH), Friedrich‐Alexander‐University of Erlangen-Nuremberg (FAU), National Cluster of Excellence “Medical Technologies - Medical Valley EMN”, Bavaria, Germany; School of Public Health, Universiy of Montreal & LASER Analytica, 1405 TransCanada Highway, Suite 310, Montréal, QC H9P 2V9 Canada

**Keywords:** Multi-criteria decision analysis, Decision-making, Health economics, Innovation planning, Stakeholder involvement

## Abstract

**Background:**

The diffusion of health technologies from translational research to reimbursement depends on several factors included the results of health economic analysis. Recent research identified several flaws in health economic concepts. Additionally, the heterogeneous viewpoints of participating stakeholders are rarely systematically addressed in current decision-making. Multi-criteria Decision Analysis (MCDA) provides an opportunity to tackle these issues. The objective of this study was to review applications of MCDA methods in decisions addressing the trade-off between costs and benefits.

**Methods:**

Using basic steps of the PRISMA guidelines, a systematic review of the healthcare literature was performed to identify original research articles from January 1990 to April 2014. Medline, PubMed, Springer Link and specific journals were searched. Using predefined categories, bibliographic records were systematically extracted regarding the type of policy applications, MCDA methodology, criteria used and their definitions.

**Results:**

22 studies were included in the analysis. 15 studies (68 %) used direct MCDA approaches and seven studies (32 %) used preference elicitation approaches. Four studies (19 %) focused on technologies in the early innovation process. The majority (18 studies - 81 %) examined reimbursement decisions. Decision criteria used in studies were obtained from the literature research and context-specific studies, expert opinions, and group discussions. The number of criteria ranged between three up to 15. The most frequently used criteria were health outcomes (73 %), disease impact (59 %), and implementation of the intervention (40 %). Economic criteria included cost-effectiveness criteria (14 studies, 64 %), and total costs/budget impact of an intervention (eight studies, 36 %). The process of including economic aspects is very different among studies. Some studies directly compare costs with other criteria while some include economic consideration in a second step.

**Conclusions:**

In early innovation processes, MCDA can provide information about stakeholder preferences as well as evidence needs in further development. However, only a minority of these studies include economic features due to the limited evidence. The most important economic criterion cost-effectiveness should not be included from a technical perspective as it is already a composite of costs and benefit. There is a significant lack of consensus in methodology employed by the various studies which highlights the need for guidance on application of MCDA at specific phases of an innovation.

**Electronic supplementary material:**

The online version of this article (doi:10.1186/s12913-015-0930-0) contains supplementary material, which is available to authorized users.

## Background

The market for health services and products is is distinct from other markets in many different ways. The demand for health services and products is largely decoupled from prices and customer preferences. Importantly, reimbursement decisions of public health care regulate access and usage of new health technologies [1]. Consequently, these decisions are the bottle neck for medical innovation in many countries with both economic and social implications [2, 3].

Currently, health policy decision-making in many countries is based on health economic concepts. Simultaneously, manufacturers increasingly use health economic tools [4–8] to assess investment decisions in the development process of medical technologies. The rationale behind these concepts is to compare the costs and the medical benefit of medical technologies. The most prominent Quality Adjusted Life Years (QALY) concept provides an estimate to relate the gain in quality of life and life years versus the associated costs of the medical technology. The advantage of a single estimate is to compare benefit and costs of different technologies across different therapeutic areas as shown by the large number of published studies based on QALYs. QALYs are widely used by HTA agencies, academia and industry because it is assumed as an objective measure to compare technologies.

Nevertheless, several studies identified flaws in major features of the QALY concept [9, 10]. A recent survey on medical utility disapproved the theoretical assumptions of QALY due to inconsistent preferences [11]. In practical application, different utility assessment methods to assess quality of life result in different QALY estimates [12, 13]. For decision-making, Richardson pointed out that the public would strongly disagree with the only use of QALY to allocate health resources [14]. A reason for this disagreement is that the benefit of health technologies is so diverse. Other aspects can confound the simple trade-off in QALYs. Apart from costs and medical benefit, many other aspects e.g. severity of disease affect the decision about medical technologies. Additionally, stakeholders have different perspectives on the diverse benefits of medical innovation [10]. As health technologies are getting more and more complex, the understanding of stakeholders on the value of these technologies diverges further [15]. Such issues question the methodological basis of trade-offs in current health policy decision-making.

Decision tools that can systematically integrate costs and benefits of medical innovations from multiple perspectives would therefore benefit all stakeholder including patients, payers and the industry. Multi-criteria decision analysis (MCDA) offers an opportunity to address this trade-off. In 1976, Keeney and Raiffa define MCDA as “an extension of decision theory that covers any decision with multiple objectives [16].” Belton and Steward describe MCDA as “an umbrella term to describe a collection of formal approaches which seek to take explicit account of multiple criteria in helping individuals or groups explore decisions that matter” [17]. As outlined by these definitions, MCDA can describe a broad range of methods. The common key aspects are the separation of a decision problem into different mutually independent criteria, the quantification of these criteria and the final aggregation to a value estimate. For medical innovation, MCDA can take different stakeholders’ preferences into account by separating the considerations on the importance of decision criteria, the evaluation of the performance of health interventions and the evidence on which such evaluation is based [18].

The objective of this study is to review applications of MCDA in decisions addressing the trade-off between costs and benefits, within the development phase and market access of health technologies. By providing an overview of published MCDA applications, this study informs potential users how MCDA can support decision problems in different decision environments, thus tackling an important step for theory, policy and practice as well as future research.

## Methods

### Eligibility criteria

The objective and the search strategy were established by using the MIP Scheme. This scheme consists of the parameters methodology, issues and participants (Methodology = MCDA, Issues = Research, development and reimbursement decisions, Participants = Manufacturers, hospital manager, health care provider, health policy makers) [19]. MIP is suitable for our research question because health economic MCDA studies are based on multiple interventions, outcomes, participants, and settings [20]. The search strategy was performed using basic steps of the PRISMA Guidelines (see PRISMA checklist in additional file 2) [21].

### Information sources and search

Medline, PubMed, Springer Link and specific journals, which deal about health economic decision-making, Value in Health, Health Affairs, Medical Decision Making, Patient, Cost Effectiveness and Resource Allocation and Pharmacoeconomics were searched from January 1990 to April 2014 [22]. According to the MIP Scheme, specific keywords focused on the methodology to keep the search as sensitive as possible. Issues and participants were included in the study selection process (Table 1). Accordingly, the following search terms were used: “MCDA”, “multi-criteria decision analysis” as well as certain methodologies: “direct weighting”, “balance sheets”, “the even swap method”, “ordinal methods”, “goal programming”, “multi attribute utility analysis and the “analytic hierarchy process”, “AHP”, “ANP”, “Discrete Choice Experiment” and “Conjoint Analysis”. The keywords were combined and adapted to each database. Additional articles were found in the references and citations of the retrieved articles.

**Table 1:** Study selection criteria

No	Category	Criteria
1	Year of release	1990- April 2014
2	Kinds of interventions	All kinds of medical interventions and technologies (no diseases)
3	Innovation process	Investment decision, prioritization of new technologies, HTA, reimbursement
4	Criteria	Studies including costs, economic analysis (should go beyond safety analysis to solve trade-off between costs and health)
5	MCDA Methodology	Original research about MCDA
6	Active stakeholder involvement	Manufacturers, hospital manager, health care provider, health policy makers
7	Source of publication	Peer reviewed journals
8	Language	English, German

### Study selection

The title and abstract of all articles identified by the database searches were reviewed. Articles meeting initial inclusion criteria (Table 1) were retrieved and examined more closely in collaboration with a second researcher (CKR) until consensus was reached. If both researchers did not agree, a third researcher was involved (CNI). In agreement with the objectives of this study, reviewing applications of MCDA methods in decisions addressing the trade-off between costs and health benefit, only studies in which the MCDA process included economic aspects were included. The quality of research papers was evaluated by checking for an adequate description of the theoretical framework, background, and methodology [23]. Research articles meeting inclusion criteria were appraised for methodological quality. The studies were required to be described comprehensively with clear description of the methods used, criteria selection, weighting, and scoring.

### Data collection process and data items

Each study was described in the data extraction form to highlight heterogeneity between studies. Firstly, information about the decision context was analyzed. This includes information about participating stakeholders and the decision problem. Secondly, the methodology and thirdly, the decision criteria were extracted, as shown in Table 2. The methodological approach of Guindo et al. was adapted to assess the included criteria. Criteria were clustered in several subgroups and quantitative measures were provided [24]. Criteria used in the Analytic Hierarchy Process (AHP) are hierarchically structured. Therefore, only the main criteria of the hierarchy were counted and sorted into the subgroups. The data extraction form was tested on a sample of studies before full data extraction began.

**Table 2:** Classification of used criteria [24]

Categories of classification system	Number of criteria	Number of studies	Terms used in articles
Health outcomes and benefits of interventions	12	16	Health effects [26], health gain (with 4 sub criteria: life expectancy, quality of life, burden of treatment, prevalence) [34], improvement of efficacy/effectiveness [36, 37, 39, 40], improvement of safety & tolerability [36, 37, 39, 40], improvement of patient reported outcome [36, 37, 39, 40], health benefit [43] , effectiveness [30, 38, 41, 42, 45, 46], patient comfort [30], safety [30, 33, 46], health-related quality of life [31, 41], complications during surgery [31], program outcome [32],
Type of health benefit	4	9	Individual health benefits [25, 27, 46], public benefits [29], public health interest [36, 37, 39, 40], type of medical service [36, 37, 39, 40, 42]
Impact of the disease targeted by intervention	10	13	Severity of disease [25–28, 35–37, 39, 40, 45], number of potential beneficiaries [25, 27, 28], size of population affected by disease [36, 37, 39, 40, 45], age of target group [25–28, 35], socioeconomic group [35], equity benefit [43], target groups of interventions [42], gender of target groups [42], eligible population [44], distribution of benefits [44]
Therapeutic context of intervention	4	5	Clinical guidelines [36, 37, 39, 40], comparative interventions limitations [36, 37, 39, 40], need [41], prevention [41]
Economic Impact	12	22	Costs [30, 31, 33], poverty reduction [25–28], cost-effectiveness [25–28, 35–37, 39, 40, 46], total budget impact to health system [26, 36–40, 43, 45], costs of care [34], marketability [29], Impact on other spending [36, 37, 39, 40], program infrastructure [32], program outcome [32], Incremental cost-effectiveness [44, 45], affordability [44, 46]
Quality and uncertainty of evidence	5	8	Adherence to requirements of decision making body [36], completeness and consistency of reporting evidence [36, 37, 39, 40], relevance and validity of evidence [36, 37, 39, 40], quality of evidence [42, 43, 46], certainty [44]
Implementation complexity of intervention	10	9	Technology applicability [29], system capacity and appropriate use of intervention [37, 40], technical feasibility [38], practical feasibility [38], information follow-up in time [38], clinical factors [33], biomedical engineering [33] , process [32, 41], variation in practice [45], technical complexity [46]
Priorities, fairness and ethics	11	7	Utility [37, 40], efficiency [37, 40], fairness [37, 40], ‘X-factors’ [43], ethical acceptability [38] access and equity [41], priorities [41], equity/ethical and social implication [45], geographical coverage [46], accessibility [46]
Overall context	10	7	Stakeholder pressure [37, 40] , political context [37, 40], ‘X-factors’ [43], impact on heath education [38], Impact on future decisions [39], relationship with pathology providers [39], impact on screening intervals [39], patient expectation [39], program infrastructure [32], acceptability [46]

### Synthesis of results

After completing the data extraction, a narrative synthesis was compiled according to the objective regarding the decision context and applied MCDA methodologies. For the decision context, study characteristics were reported regarding the countries of origin, intervention characteristics, the level of decision-making, stakeholder involvement, the level of innovation and evidence characteristics. A descriptive analysis of decision criteria was performed separated into benefit criteria and cost criteria. To provide a technical overview, the applied methodologies were systematically decomposed to identify weighting and scoring techniques. Additionally, stakeholder feedback on the methodology was reported.

## Results

### Literature search

**Fig. 1:**
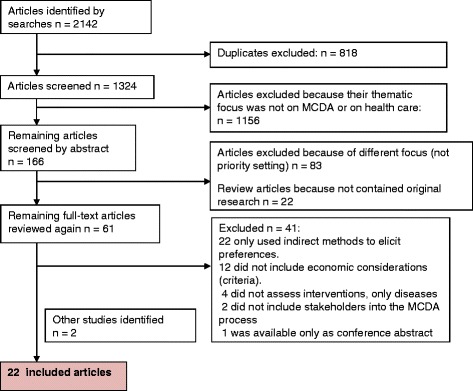
Literature selection flow diagram

2142 papers were retrieved (Fig. 1) and 1974 were excluded because they were duplicates or not focused on MCDA or health care. Of the remaining 166 articles, 61 articles were selected for review by two authors (PWA, CKR) following the criteria in Table 1. Finally, 22 papers were selected for the literature synthesis [25–46]. The reasons for exclusions are outlined in Fig. 1.

### Decision context of the MCDA studies

#### Countries

Table 3 provides descriptive statistics on the included studies. The 22 studies are from 12 countries, mostly high- and middle-income (14 studies): Netherlands (4), Korea (2), Canada (3), UK (2), US (1), France (1), Israel (1), South Africa (1) and Thailand (2). The four other studies were from low-income countries: Ghana (2), Nepal (1) and Ivory Coast (1). Most of the evidence originates from countries where cost-effectiveness approaches are already in use as part of funding allocation. These countries include developed countries like the Netherlands [47] and Canada [36, 37, 40], where policy-makers already consider multiple criteria, but also developing countries like Ghana, where several DCE studies were reported [26, 28]. Studies about early innovation are all from high-income countries because the innovators are located there [29–31, 34].

**Table 3:** Descriptive statistics of included studies

Article	Year	Country	Type of HTA	Methodology
Baeten [25]	2010	International (Netherlands, US, UK)	Mainstream	DCE
Baltussen [26]	2005	Ghana	Mainstream	DCE
Baltussen [27]	2007	Nepal	Mainstream	DCE
Bots [34]	1995	The Netherlands	Very early	SMART (simple attribute rating technique)
Cho [29]	2000	Korea	Very early	AHP
Diaby [35]	2011	Ivory Coast	Mainstream	DCE
Goetghebeur [36]	2012	Canada	Mainstream	Direct weighting, on 5-point scale
Goetghebeur [37]	2010	Canada	Mainstream	Direct weighting, on 5-point scale
Golan [43]	2012	Israel	Mainstream	PAPRIKA (Potentially All Pairwise RanKing of all possible Alternatives)
Hilgerink [30]	2011	The Netherlands	Early	AHP
Hummel [31]	2012	The Netherlands	Early	AHP
Jehu- Appiah [28]	2008	Ghana	Mainstream	DCE
Le Gale [38]	1990	France	Mainstream	Direct weighing and outranking (ELECTRE 1S Model)
Miot [39]	2011	South Africa	Mainstream	Direct weighting, on 5-point scale
Marsh [44]	2012	UK	Mainstream	DCE
Shin [32]	2008	South Korea	Mainstream	AHP
Sloane [33]	2003	US	Mainstream	AHP
Tony [40]	2010	Canada	Mainstream	Direct weighting, on 5-point scale
Venhorst [46]	2014	Netherlands	Mainstream	Direct weighting
Wilson [41]	2006	UK	Mainstream	Weighted benefit score (WBS)
Youngkong [42]	2011	Thailand	Mainstream	DCE with deliberation process
Youngkong [45]	2012	Thailand	Mainstream	Direct weighting with consideration of DCE results

#### Interventions

The examined MCDA studies assessed a broad range of medical interventions at different stages of innovation. These include medical devices (neonatal ventilators, breast cancer screening technology), drugs (growth hormone, tramadol) and different service programs (breast cancer screening and treatment, prevention programs, surgical treatment lung health, liquid-based cytology for cervical cancer screening, free vaccination service, and HIV interventions). Some MCDA studies only assessed one or a few interventions, whereas other studies rank up to 56 interventions.

#### Level of decision-making

The studies were conducted at different levels of decision-making. Two studies applied MCDA for decision-making on an international level. The majority of 14 studies examined decision-making on a national level. Three studies assessed regional decision problems and one study was conducted in a hospital setting. Two further studies assessed the development of new products on the level of manufacturers. Eight studies resulted in implementation: an official committee considered the MCDA results in their final decisions. Other studies were conducted in an explorative manner.

#### Stakeholders

**Fig. 2:**
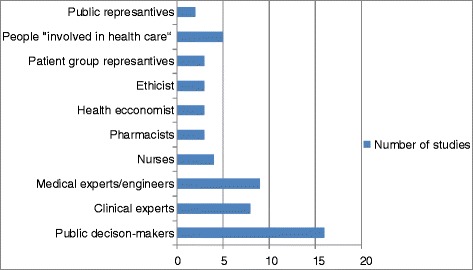
Stakeholder involvement

In terms of stakeholder involvement, Fig. 2 illustrates that health policy decision-makers were the most strongly involved groups in 16 studies (73 %). Patient involvement was rare (3 studies, 14 %).

#### Level of innovation

This review divided the medical product development into three levels adapted from Izjerman et al. [7]. Accordingly, very early HTA is applied on the stage of basic research. Early HTA covers the stage of translational research whereas main stream HTA addresses clinical research and market access. Two studies focused on the very early HTA and the question of which kind of technology should be supported [29, 34]. Two studies examined the development and specifications of a certain innovation (early HTA) [30, 31]. Three of those four studies used an AHP. The majority of 17 studies examined fully developed technologies on the level of market access and reimbursement decision (Main stream HTA).

#### Evidence

The reviewed studies used evidence from different sources. Table 4 groups them into literature reviews, expert opinions, data obtained from health care system/decision-making bodies/manufactures and health economic modeling. One study used fictitious data [41]. Another study did not state the underlying evidence [45]. There were large differences in the level of analysis performed on the evidence. Most studies did not describe a systematic approach for reviewing the evidence. Only studies based on the EVIDEM (Evidence Based Decision Making) framework, which requires providing synthesized evidence for each decision criterion, developed a full HTA report made of 14 to 69 references including evaluation of the quality of the evidence. The earlier the MCDA was conducted, the more expert opinions were included.

**Table 4:** Evidence used by studies

Evidence	Literature review	Expert opinions	Data obtained from health care system/decision-making bodies/manufactures	Health economic modeling
**Number of studies**	14 [25–28, 30–32, 35–37, 39, 40, 42, 44]	10 [26–29, 31–34, 38, 46]	7 [30, 33–35, 38, 39, 43]	2 [31, 44]

#### Decision criteria

The surveyed studies obtained MCDA decision criteria from literature research and context-specific studies, experts in the field, and group discussions with stakeholders. Some studies used these methods simultaneously. The selection of criteria depended on the country-specific decision context, the diffusion level, and the intervention type. The number of criteria ranged between three and 15, up to 25 if sub-criteria of AHP were counted [32]. An analysis of all criteria is summarized in Table 2.

#### Benefit criteria

The most frequently used criteria described health outcomes, disease impact and implementation of the intervention. Twelve different criteria measuring health outcomes and benefits were used in 16 studies (73 %). The impact of the targeted disease was used by 13 studies (59 %) in ten different terms. Implementation complexity of intervention and the type of health benefit were included in eight studies (41 %).

#### Cost criteria

As a study selection criteria (defined in Table 1), economic criteria were used in all included studies. The number of economic criteria in the assessed studies ranged between one and three. These criteria included cost-effectiveness (14 studies, 64 %), and total costs/budget impact of an intervention (eight studies, 36 %). There is a widespread heterogeneity among the process of including economic criteria. Four studies considered budget impact and impact on other spending simultaneously to cost-effectiveness [36, 37, 39, 40]. Economic considerations in different criteria can overlap in certain MCDA models. The criterion “cost-effectiveness” covered the costs of a technology whereas the criterion “age of target group” included economic considerations regarding the working part of society [27]. An AHP study included costs in several sub criteria like “Investment resources for infrastructure”, “governmental budget” and “economical satisfaction” [32] Four studies considered the budget impact of an intervention in a second step after conducting the MCDA [35, 41, 42, 45]. Diaby [35] assessed cost-effectiveness in a first step. In a second step, budget impact analysis was used to define reimbursement threshold per patients until the financial threshold is exhausted. In another study, costs were included by calculating costs per point in a second step after weighting [41]. In a DCE study, cost-effectiveness was added into the deliberative process after the DCE [42]. Another DCE study assessed effectiveness and economic impact of household expenditures in the first assessment step, value for money (ICER) and budget impact in the second assessment step [45].

In early innovation studies, the perspective on economic considerations was different due to the absence of economic evidence. Two studies describing an AHP considered costs in several sub-criteria [29, 32]. Costs were a main criterion divided into investment in materials and treatment costs [31]. Costs were one of four criteria with the sub criteria: scan duration, manpower, price and peripheral equipment. However, the authors suggest that cost should be used as a main criterion without sub-criteria because of several overlaps in the hierarchical structure [30]. Bots et. al calculated changes in the cost of care to determine cost-effectiveness [34].

### Operationalization of MCDA in the examined studies

MCDA consists of several steps including the assignment of criteria weights, the assignment of performance scores and the derivation of the total scores. Some studies also conducted a sensitivity analysis or a deliberative process. The examined studies performed these steps in various ways and used various quantitative techniques for scoring and weighting processes.

#### Weighting

Seven studies conducted a DCE [25–28, 35, 42, 44]. In DCE studies, participations had to choose their preferred intervention from sets of hypothetical scenarios. These interventions are described by different criteria over a range of levels. Finally, critria levels weights are calculated.

All other studies used MCDA approach that allows participants to directly assess the criteria. Within this group, the AHP was most prevalent (five studies) [29–33]. Participants directly compare certain criteria via trade-offs on a scale from one to nine. Weights can be calculated via the right eigenvector.

Six studies were based on a direct weighting approach [36, 37, 39, 40, 45, 46]. Five studies applied direct weighting on a one to five scale [36, 37, 39, 40, 46]. One study considered the results of a DCE in a deliberative process. As result, six criteria received equal weights [45]. One study applied the SMART (simple attribute rating technique) approach by using direct weights with a scale from 0 to100 [34].

Another study used the outranking approaches of ELECTRE (ELimination and Choice Expressing Reality) in combination with direct weighting. Direct weighting was performed for five criteria on a 5-point scale, whereas two criteria (costs and effectiveness) directly included into the outranking model [38]. In the study using PAPRIKA (Potentially All Pairwise RanKing of all possible Alternatives) participants did trade-offs between hypothetical technologies. Afterwards, a software program (1000minds) calculates weights for each criterion and level [43]. Another study applied weighted benefit scores (WBS) performed the weighting exercise by allocating 100 points among criteria [41].

#### Scoring

Weighting in DCE studies was performed by using composite league tables whereas one study explicity added a deliberative discussion about the result [42]. One DCE study obtained combined weights and scores as utilitly scores from a deicison model [44]. Again, AHP studies applied trade-off rating as described above to obtain scores. Two studies about early HTA calculated scores from expert opinions [30] and decision trees [31].

The SMART study used a scale from one to five to score the evidence [34]. The process was similar y in another study. Six criteria were scored on a scale of one to five. Two further criteria were considered as numerical measures in the final discussion [45]. The four studies based on the EVIDEM methodology applied direct weighting on a scale from one to four [36, 37, 39, 40]. Scoring was performed on a scale from zero to two in another study [46]. For the outranking study, experts’ view on criteria performance guided the outranking process (ELECTRE 1S) [38]. The performance was directly scored on a scale from one to ten in another study [41]. The finals scores were combined with the costs of options, which resulted in a cost/score ratio. One study stated that the first author judged the performance levels of the assessed technologies [43].

#### Stakeholder feedback on methodology

Stakeholder feedback on the MCDA approach was described in 12 studies. One study reported that the hierarchical structure of AHP seemed too complex to participants. The high number of evaluated alternatives made selection even more difficult [29]. The contribution of disease specific decision criteria is limited if several interventions for the same disease are assessed [25]. Scoring exercises were difficult in several studies. The number of criteria levels needs to be sufficient to represent the real world [48]. In DCE studies, scoring of criteria with two levels was sometimes not sufficient. However, adding more levels would have rendered the discrete choices for respondents more complex [26, 28, 35, 42].

## Discussion

The objective of this study was to review applications of MCDA methods in decisions addressing the trade-off between costs and benefits. This review identified important points relating to implementation of MCDA approaches.

### Methodological shortcomings

There seems to be remarkable lack of consistency in methodology employed by the various studies that undertook the MCDA. There was no reasonable process to make clear why different authors decided to use a certain methodology. The selection of criteria was more systematic but still very different across studies. This resulted in different numbers and sort of criteria in the examined studies. Additionally, the scaling of performance scores raises questions about the methodological validity of these scales.

In contrast to these shortcomings, the stakeholder feedback on MCDA highlights the potential value of the methodology for decision-making. Therefore, we developed some recommendations for the technical implementation of MCDA in decision-making. Determinants of quality were identified regarding the technical aspects of decision criteria, the scaling of criteria to measure performance, the weighting method and the underlying evidence.

### Defining decision criteria

MCDA can bridge criteria and views that are challenging to compare, as shown in the specific bioethical context [49]. Choosing and clustering the right criteria requires active and direct involvement of stakeholders to avoid irrelevant or overlapping criteria [45]. The decision criteria should be technically robust regarding potential overlaps [37]. Every criterion should focus on a single aspect of the decision to avoid double-counting [50]. Double counting means that the same effect impacts more than one criterion in the performance matrix. For example, even though cost-effectiveness was one of six decision criteria in a study, the criterion “age of target group” included economic considerations regarding the working part of society [27]. The implicit consideration of economics in different criteria can bias the results. For health policy decision-making, another important criterion for double counting is the severity of disease. This criterion can impact several other criteria e.g. economics, effectiveness, ethics. Therefore, the explicit structuring of criteria is important. Checking for independent preferences can support the detection of double-counting effects. A deliberative process can increase the legitimacy of MCDA criteria selection [42, 51].

### Issues on economic criteria

Few studies have suggested the use of separate criteria describing costs and medical effects instead of the mixed criterion cost-effectiveness [36, 37]. This makes decision criteria more transparent and understandable e.g. absolute costs of an intervention as an easy understandable measure. Still, cost-effectiveness is the most important economic criterion in the examined studies which shows a lack of awareness regarding double counting. Several studies used more than one economic criterion indicating that the absolute costs of an intervention should be compared to the impact on the available budget.

The inclusion of economic aspects in decision-making by MCDA can be very different. Most studies compared economic criteria with other criteria in one step. In contrast, some studies considered economic criteria like budget impact in a second step. This procedure can feed a deliberative process [42, 45] or a mathematical model to calculate the reimbursement threshold [35, 41].

In early innovation studies, economic parameters were simple due to the absence of health economic evidence. For example, economics for the prospective usage of photoacoustic imaging for breast cancer diagnosis was assessed by the criteria “price”, “manpower”, “scan time” and “peripheral equipment” [30]. In contrast, a study about new surgery in adolescent idiopathic scoliosis included cost parameters into a decision tree model to estimate total costs [31]. This raises the question whether health economic analysis should be performed in early innovation by taking uncertainty into account or to assess only those simple parameters.

### Scaling of criteria

A well-defined scale for every decision criterion should represent the performance of the assessed alternatives. Doing so, judgments on well-defined scales are proportional and consistent with the increase in real world performance. Constructing different levels of performance for decision parameter is not a trivial task [35]. The discriminatory power of MCDA can be decreased if the scaling system and thresholds are not inadequately constructed [45]. Most of the assessed studies used rating scales with five or less categories. However, psychological research showed that scales with more categories (i.e. 10, 101) support users in expressing their feelings. In contrast, rating scales with only a few categories support quick ratings. For most situations, rating scales with seven, nine or ten categories are most appropriate taking all pro and cons into account [52]. Scaling of criteria should take reliability, discriminatory power and preferences of participants into account. A sensitivity analysis can help to determine the uncertainty from scaling effects e.g. whether more scaling levels contribute to a more exact result [35, 41]. This can be used to iteratively improve the MCDA model.

According to the value function approach, direct rating of the decision criteria by participants can improve the awareness in decision-making [34, 36, 37, 39, 40]. This approach reflects the need for explicit statements about the importance of criteria. Doing so, the importance of criteria needs definitions among different dimensions [53]. Firstly, direct weighting provides a relationship between the importance of two criteria (e.g. value of criterion A = 2 vs. value of criterion B = 4). Secondly, trade-off weights between criteria need to be compared taking the ranges on the criteria scales into account. This is covered by the scoring exercise which is required of any MCDA approach that was assessed in this review. Thirdly, the starting point for assessing value trade-offs needs consideration, in particular if preferences are not linear. A linear relationship between performance measure and scale is not always appropriate even if measures are constructed in a linear way [7]. To cover all three dimensions of importance, MCDA methods like PROMETHEE (Preference Ranking Organisation Method for Enrichment Evaluation) enables the clear definition of preference functions [54].

### Selection of MCDA method

The selection of the MCDA method can affect the uncertainty of decision-making. Different MCDA approaches vary in structuring the decision problem (i.e. criteria selection, weighting, scoring, and calculating). This variety causes structural uncertainty [55]. Each MCDA method has particular advantages for specific phases of the innovation process [29, 34]. For example, methods like DCE are very efficient at the beginning of technology development as they show the conscious and unconscious preferences of stakeholders. If evidence around technologies is generated, direct methods can be used for decision problems about reimbursement [39]. Uncertainty around evidence can lead to wrong decisions such as reimbursement of an ineffective therapy. An advantage of MCDA is that a structured decision framework can make this uncertainty more visible within the structure. The evidence and comments of the stakeholders, involved in the studies, suggest that direct rating methods can better handle this uncertainty due to a better understanding of the methodologies [29, 35, 42]. Additionally, preference elicitation techniques cannot support larger numbers of criteria and scaling levels [25, 28, 35, 42, 44] which again increases the uncertainty within the MCDA structure. The combination of different MCDA methods for different parts of the decision, like criteria weights from DCE and evidence scores from AHP can improve the decision analysis [25]. Combining different approaches requires assessing the transferability of the stepwise results. Handling of MCDA approaches may vary across stakeholder groups [29, 37]. For instance, applying MCDA can be challenging for laypeople and patients [42, 45]. The evidence suggests that the cognitive burden of direct weighting methods is lower in comparison to AHP and DCE [29, 35, 42]. Pragmatic MCDA means to provide simple and flexible approaches which are easy to understand by users with little experience. Doing so, the needs of decision-makers should be carefully balanced with the resource requirements as well as the theoretical requirements of certain MCDA methodologies. Feasibility and flexibility are important aspects to ensure acceptability of these approaches [36, 37, 39, 40].

### Stakeholder involvement

Awareness about the selection of decision criteria, their importance [56] and the decision perspective are critical parts of MCDA from an ethical viewpoint. Stakeholders have to reflect on their own priorities and rationale to elucidate their decision-making. Patient involvement was limited and reported in only 14 % (3 studies), although they represent the ultimate decision makers on final acceptance of a healthcare intervention. Inhalable insulin is an example for innovation failure because of missing user acceptance in the final phase of the innovation process [57]. A consistent mix of stakeholders across all stages of innovation can ensure that all important needs are addressed. The separation of weighting and scoring by different stakeholders can result in disagreement [34]. Still, combining many stakeholder perspectives helps to identify and possibly resolve differences across perspectives. An essential aspect is to identify the “right” representativeness of a certain stakeholder group [45] as well as the involvement of the “right” stakeholder group. Indeed, the question, which perspective (their own or another e.g. society) participants should take into account, is not trivial [37]. Daniel’s ethical framework of “accountability for reasonableness “ is a widely-agreed rationale regarding fair decision-making [58]. According to this framework, reimbursement decisions should be based on reasons and criteria which reflect society’s values [59]. However, the consensus of experts does not guarantee that choices are representative of a societal viewpoint [38, 44]. MCDA approaches can help make these issues more explicit and thus provide some methodological basis to feed the ongoing debate in society about reimbursement decisions.

### Evidence needs

Incomplete evidence for the assessed alternatives increases uncertainty around decisions. Pragmatic MCDA bridging HTA methodology with MCDA principles can help clarify these issues in a systematic manner [36, 37, 39, 40]. Still, missing data and a time-consuming workload make existing approaches insufficient [45]. In contrast to mainstream HTA, the evidence in the development of a technology is even more based on expert opinion or adaption of evidence from other technologies [29–31, 34]. Most reviewed studies related to early innovation lack economic considerations because of missing evidence. Finally, missing evidence can put new technologies at disadvantage in prioritization decisions [44]. Expert judgments could replace clinical evidence but the selection of experts can affect the results [31]. Evidence can also be obtained by early health economic modeling [31, 44]. There is the potential for synergies between evidence generation and MCDA. All MCDA require the involvement of stakeholder groups to some extent. Different stakeholder groups can participate in the MCDA decision process and thus improve the communication of their preferences and perspectives in a systematic way [18, 60]. Values and preferences of stakeholders are essential to integrate different decision criteria and to come up with a decision. For example, AHP supports consensus finding in groups by calculating an inconsistency ratio after the scoring exercise. This can be the basis for a deliberative discussion to refine ratings. In terms of pragmatic MCDA, stakeholder can contribute to the selection of decision criteria and the selection of MCDA techniques to evaluate these criteria. Simultaneously, experts and stakeholders can support the evidence search and synthesis, the validation of health economic models, as well as the MCDA process. Such combination can contributes to a transparent analytic process and results in a more comprehensive understanding of the technology in every phase of innovation. MCDA can facilitate an early dialogue between manufacturers, regulators and HTA agencies about evidence needs. This collaboration can help patients by faster access to treatments as well as manufacturers for more efficient development of new technologies.

### Limitations of this study

This study should be considered in light of its limitations. We only included studies that used economic criteria, a component that was missing in a significant part of the literature. As there is no clear definition of MCDA, we only included studies which mentioned MCDA or a certain described MCDA method. However, other studies may use MCDA methods without explicitly stating it. The results reported in the indexed literature could be systematically different from those presented in the grey literature such as government reports and white papers [61]. Further research should focus on how MCDA can contribute to an efficient innovation process as well as the dynamics of changing conditions regarding new evidence and its impact on decision-making. Development of guidelines on the choice of a particular approach to use at specific phases of the innovation process would provide value for further development in the field. The comparability and the usefulness of the final MCDA outputs also requires some further research. Baltussen suggest using the final result of an MCDA as recommendation, not as formal list of priorities (60). Another line of research is identifying the implementation and administrative workloads of MCDA in comparison to existing approaches, and the overall benefit in decision-making.

## Conclusions

This study reveals that MCDA facilitates the trade-off between costs and benefit in different decision settings but cannot replace the reflection required for good decisions. MCDA can however increase the transparency, quality and consistency of decisions. In early innovation, MCDA can provide information about stakeholder preferences as well as evidence needs for further development. Such approaches increase the efficiency of the R&D process and facilitate access to most beneficial innovations. For HTA, MCDA allows a more nuanced analysis in different settings and different countries by explicitly structuring decision criteria and providing a methodological framework for decision-makers to address the conflict between costs and medical benefit. Further research is needed to define guidelines about the conditions of MCDA at specific phases of an innovation. These should address the appropriateness of certain MCDA methods, the robustness of models regarding potential criteria overlaps, performance scales and opportunities for operationalization. Scaling of criteria’s performance, like severity of disease, needs careful considerations particularly regarding a sufficient discriminatory power. MCDA supports understanding the rationale behind decision-making processes in complex health care investments, and the constraints of sustainability, efficiency and equity that healthcare systems are facing.

## Additional files

Additional file 1:
**Search strategy and data extraction forms.**
Additional file 2:
**PRISMA 2009 checklist.**

## Abbreviations

AHP: Analytic Hierarchy Process
CA: Conjoint analysis
DCE: Discrete choice experiments
ELECTRE: ELimination and choice expressing reality
EMA: European medicines agency
EVIDEM: Evidence based decision making
FDA: Food and drug administration
HTA: Health technology assessment
IQWIG: Institute for Quality and Efficiency in Health Care
MCDA: Multi-criteria decision analysis
NICE: National Institute for Health and Care Excellence
PAPRIKA: Potentially All Pairwise RanKing of all possible Alternatives
PRISMA: Preferred reporting items for systematic reviews and meta-analyses
QALY: Quality adjusted life years
SMART: Simple attribute rating technique
WBS: Weighted benefit scores
