# Supplementary material for: Balancing costs and benefits at different stages of medical innovation: a systematic review of Multi-criteria decision analysis (MCDA)
Source: BMC Health Serv Res. 2015 Jul 9;15:262. doi: 10.1186/s12913-015-0930-0 (PMC4495941; doi:10.1186/s12913-015-0930-0)
Supplement: Additional file 1: — Search strategy and data extraction forms. [file 12913_2015_930_MOESM1_ESM.docx]

**Additional file 1**

**Search in different databases**

| **DATABASES** | | | | | | |
| --- | --- | --- | --- | --- | --- | --- |
|  | “MCDA” | “Multi-criteria decision analysis” | “DCE” AND “Discrete Choice” | “AHP” OR “ANP” AND (health care OR intervention) | “Direct Weighting” | “Conjoint analysis” |
| Medline | 101 | 1 | 95 | 188 (analytic hierarchy process) | 52 | 301 |
| Pubmed | 167 | 130 | 114 | 150 | 25 (AND health care) | 263 (AND health care) |
| SpringerLinks (Filter: Medicine) | 22 | 23 | 89 |  |  |  |
| Cochrane Library | 2 | 1 | 3 | 2 |  | 27 |
| NEJM | 0 | 0 |  |  |  |  |
| **JOURNALS** | | | | | | |
| Medical Decision Making | 4 | 12 | 10 | 28 | 2 | 85 |
| Health Affairs | 0 | 2 |  | 9 |  |  |
| Pharmacoeconomics | 2 | 3 | 83 | 5 |  |  |
| Health Policy | 3 | 7 | 13 | 3 |  | 22 |
| Patient | 6 | 8 | 10 | 0 | 61 |  |
| Value in Health | 3 | 15 | 8 | 1 |  |  |
| Cost Effectiveness and Resource Allocation | 1 | 3 | 1 | 0 |  |  |

**Example for search strategy in Pubmed:**

“MCDA” [All Fields] AND ("1990/01/01"[PDat] : "2014/04/30"[PDat])

**General description of the decision context and the methodology**

| **No** | **Decision context of MCDA** |
| --- | --- |
| 1 | Study Site(s), Year |
| 2 | Objective |
| 3 | Setting/population |
| 4 | Type of innovation assessment |
| 4 | Type of intervention |
| 5 | Participants |
| 6 | Results |
| 7 | Practical issues, social and cultural factors that may affect decision |
| 8 | Outlook and recommendation |
| 9 | Economic evaluation |
| **No** | **Process of MCDA** |
| 1 | MCDA type |
| 2 | Selection of criteria |
| 3 | Number of criteria and criteria used |
| 4 | Weighting method |
| 5 | Obtaining the evidence |
| 6 | Scoring method |
| 7 | MCDA Model |
| 8 | Practical issues, limitations that may affect MCDA |
| **No** | **Classification of criteria** |

**Reduction of uncertainty in decision-making**

**Data extraction form 1: Decision context**

| **First author** | **Study Site(s), Year** | **Objective** | **Type of innovation assessment** | **Population/Setting** | **Type of intervention** | **Participants** |
| --- | --- | --- | --- | --- | --- | --- |
| **Baeten [1]** | International (Netherlands, US, UK), 2010 | To compare three equity-efficiency trade-off methods | Mainstream | European population | Breast cancer interventions (Screening and treatment) | Policy-makers form Europe and North America [2] |
| **Baltussen [3]** | Ghana, 2005 | To determine the importance of criteria in priority decision-making of health interventions | Mainstream | Ghanaian population (not explicitilly stated) | Interventions to reduce risks of disease | 30 health policy makers |
| **Baltussen [4]** | Nepal, 2007 | To identify, weight and assess criteria for priority setting in Nepal | Mainstream | Nepalese population | Implemantation of a Lung Health programm (PAL) (33 interventions related to child and maternal health, TB and HIV) | To determine criteria: seven policy makers and people otherwise involved in regional health care programmes Final DCE: 66 respondents (health professionals/ public health experts) |
| **Bots[5]** | Netherlands, 1995 | To define interventions that have high potential for efficiency improvements | Very early | Dutch health care insurance board | Types of medical care and organization of care | Various key specialists from the major health research institutions including one key director of the ministry of health |
| **Cho [6]** | Korea, 2000 | Korean medical device and material industry | Very early | to assess selected medical devices and materials for grants by the Korean Ministry of Health and Welfare | 88 medical devices and materials | Twelve participants (eight medical experts and four medical engineering experts) |
| **Diaby [7]** | Ivory Coast, 2011 | exploration study with the purpose of developing a method for formulary listing | Mainstream | Public health coverage: 70% of the medical expenses, services are organized and managed by the General Mutual Benefit Fund for Civil Servants and State Employees of Coˆ te d’Ivoire (MUGEFCI; Mutuelle Generale des Fonctionnaireset Agents de l’Etat de Co te d’Ivoire) | drugs indicated for hypertension, diabetesmellitus, asthma, malaria, fungal infections, upper respiratory tract infections, urinary tract infections | Three pharmacologists, a specialist in public health and health economics from MUGEFCI, two pharmacists |
| **Goetghebeur [8]** | Canada, 2012 | A field-test of the EVIDEM framework using medicines for various indications | Mainstream | View from the societal level | Ten medicines from 6 therapeutic fields submitted to the Canadian drug reimbursemnet advisory board between 2005 and 2007 | Thirteen of 39 invited participants: 3 health policy decision makers, 3 clinical specialists, 1 general practitioner, 2 nurses, 2 pharmacists, 2 health economists |
| **Goetghebeur [9]** | Canada, 2010 | To test the EVIDEM framework | Mainstream | View from the societal level | growth hormone (GH) for Turner syndrome (TS) | 4 academic pediatric endocrinologists, 1 ethicist, 1 nurse, 1 patient/patient group representative, 2 health economists/epidemiologists |
| **Golan [10]** | Israel, 2012 | Development and introduction of a framework for health technology prioritization | Mainstream | Population of Israel | 18 drugs for different indications | 44 professionals or researchers in health care, 5 patients ’representatives, 12 members of the general public, 13 researchers of Bioethics |
| **Hilgerink [11]** | The Netherlands, 2011 | To determine the most promising application for PA imaging in breast cancer | Early | Not applicable, intervention in development | Photoacoustic Mammoscope (PAM) against x-ray mammography and ultrasonography | 7 participants: 1 manager, 1 radiologist, 1 radiology assistant, 4 physicists with different background |
| **Hummel [12]** | The Netherlands, 2012 | To support the development of Non-fusion surgical treatment by predicting it’s health economic performance | Early | Not applicable, intervention in development | Non-fusion surgfical treatment (NFS) for Adolescent idiopatic scoliosis | 4 biomedical engineers and two orthopaedic surgeons |
| **Jehu-Appiah [13]** | Ghana, 2008 | To estimate the importance of criteria and rank order a set of interventions | Mainstream | Ghanaian population, results were used for focussing the Five year program of work (POW) “to improve health status and reduce ineuqalities in health” | Interventions for childhood diseases, communicable and noncommunicable diseases, reproductive health and injuries | 63 regional and district directors (Ghana Health Service senior management meeting) |
| **Le Gales [14]** | France, 1990 | To reach consensus about screening strategy | Mainstream | Southeastern France (Marseillei region, 1.7 Million inhabitannts) | Screening strategy for carriers of heterozygote hemoglobinopathies | 17 medical experts with different background |
| **Marsh [15]** | UK, 2012 | To develop an MCDA approach for prioritizing investments | Mainstream | Population of UK | 17 public heatlh interventions for prevention | 83 survey respondents: executive directors, directors, chief executives from health and social care |
| **Miot [16]** | South Africa 2011 | To assess the EVIDEM framework | Mainstream | Major private health plan: Discovery health (7 million lives of private health sector in total) | Liquid-based cytology for cervical cancer screening | 9 members of an clinical policy and decision-making committee, including physicians, pharmacists and nurses |
| **Shin [17]** | South Korea, 2008 | To evaluate two alternatives health care policies | Mainstream | South Korean population | Free vaccination service to children at private clinics or public health centres | 88 participants, sorted in 5 groups of experts: consultation professor group, participating institutions, implementing institutions, research groups, decision-maker group |
| **Sloane [18]** | US, 2003 | To perform a microeconomic Health Technology Assessment for the evaluation of neonatal ventilators for a hospital. | Mainstream | American Hospital with more than 500 beds in one of the top 10 US cities | Different neonatal ventilators | 2 experts: the director of the respiratory therapy department and the assistant director of the biomedical engineering department |
| **Tony [19]** | Canada, 2010 | to test EVIDEM within a drug advisory committee | Mainstream | Drug Advisory Committee of the Ontario Workplace Safety Insurance Board (WSIB), which provides healthcare benefits, to workers suffering injury or illness directly related to work. | tramadol for chronic non-cancer pain (CNCP) affecting 25% of the Canadian population | 9 members of the Drug Advisory Committee of the Ontario Workplace Safety Insurance Board (WSIB), |
| **Venhorst [20]** | Netherlands, 2014 | To prioritize breast cancer interventions in low- and middle – income countries | Mainstream | Population in low- and middle – income countries (WHO rating tool) | breast cancer interventions | 29 experts in priority settin or breast cancer policies in low- and middle income countries (epidemiologists, cancer survivors, pathologists, guideline-developers, public health specialists, radiotherapists, surgeons, researchers, managers, strategists and ethicists) |
| **Wilson [21]** | UK, 2006 | To develop and test a tool for rational and defensible decision-making | Mainstream | This case study was implemented in the decision making process of English PCT | acute service development, preventative programme, screening programme and mental health programme | 20 representatives from the local health economy, including NHS clinicians, PCT and acute trust managers, social services and the voluntary sector |
| **Youngkong [22]** | Thailand, 2012 | To experiment with the use of multi-criteria decision analysis (MCDA) to prioritise interventions in HIV/AIDS control | Mainstream | Population in Thailand | 40 HIV/AIDS interventions | 6 policy makers, 6 patient representatives, 6 community members |
| **Youngkong [23]** | Thailand, 2012 | To assess the use of MCDA regarding fair decisions in the development of the universal health coverage benefit package in Thailand | Mainstream | Population in Thailand | 17 interventions, 9 were selected for further assessment | Stakeholders involved into the Thai health care system: policymakers, health professionals, academics, patients and public representatives, industry, laypeople |

**Data extraction form 2: decision context**

| **First author** | **Results** | **Practical issues, social and cultural factors that may affect decision** | **Outlook and recommendations** | **Economic evaluation** |
| --- | --- | --- | --- | --- |
| **Baeten [1]** | Comprehensive treatment program for women below 75 years of age and treatment in stage III breast cancer were most attractive, with both an 82% selection probability, followed by screening programs for the two age groups. All approaches except the multi criteria decision analysis rank the extensive program (i.e., screening with all types treatment) as most attractive. | Disease at a lower age is an important criterion: contributing factors are the number of potential beneficiaries is higher, the low cost-efficiency, and the high net individual health benefits in the younger age group. | Not stated | Preferences of costs and effectiveness were combined in one criteria |
| **Baltussen [3]** | Cost-effectiness, severity of disease, targeting a young population were the most important criteria.  The interventions with the highest priority were for HIV/AIDS control, pneumonia and diarrhoea | Only policy makers’ preferences, which may not necessarily reflect those of the general population | Explorative analysis cannot be readily used in the priority setting process in Ghana. Further analysis should be impplemented in the local policy context and should be fed with locally meaningful information on intervention characteristics. | Preferences of costs and effectiveness were combined in one criteria. |
| **Baltussen [4]** | Highest ranking obtained interventions for TB and child health. Severity of disease and interventions that targed middle-age group were the most important criteria. | Results were in line with the one of other surveys (e.g. higher priority to interventions for people of middle age) | the priority-setting process was not embedded in the organizational context (e.g. Ministry of Health in Nepal) and its results have not been discussed with a range of stakeholders as organized, follow-up research should aim to embed the approach in that context. | Cost-effectivness was one of six criteria, but also age of target group included economic considerations of working part of society, butget-impact was not included which is also a limitation |
| **Bots[5]** | List of 31 priority issues in medical care or organization of care | Participants felt the method as coercive for example the sensitivity analysis could find changes in the ranking by changing the weights of decision makers | The ministry of health decided to implement the list for decisions about research program and medical guidelines, an update was produced in 2001 | Included changes in the cost of care to determine cost-effectiness |
| **Cho [6]** | Marketability has an importance ratio of 0.42, technology was next with 0.36 and public benefit with a ratio of 0.21. Unabsorbable suture, with a weight of 0.028, was the most important product, Intravenous cannula and central venous catheter followed, with weights of 0.027 and 0.024. | The Korea Health Industry Development Institute (KHIDI) was asked by the Minister of Health to provide a plan for financial support to small and medium firms. | The priority rankings were submitted to the evaluating team of Health and Medical Technology Planning and Evaluation Board (HPEB) of KHIDI. The results were included in the 2000 R&D support plan for health and medical technology. The priority results were referred to when budgeting $540 000 to support top 15 products of the evaluation. | The economic features of this process were part of all criteria. |
| **Diaby [7]** | The rank ordering of treatments resulted in a general preference for priority reimbursement of antimalarials, treatments for asthma and antibacterials for urinary tract infections | Of the 4 criteria, only 2 were included in the final analysis. Socioeconomic status and age was not considered to be important. | MCDA has successfully been applied to formulary listing. | Assessment of cost-effectivness, Budget impact analysis was used to define reimbursemnet threshold per patients, this can help to decide about reimbursement until the financial threshold is exhausted |
| **Goetghebeur [8]** | MCDA estimates varied on a scale from 0 to 1 between 0.42 to 0.64 for appraised drugs. | Participants had difficulties to determine their perspective (individual vs. sociatal). MCDA was felt as helpful to understand te rationale behind the decision. However, participants think that the estimates should be seen as final results. Other qualitative criteria should be added to these estimates (qualitative EVIDEM criteria were missing in this stud) | Further research about the usefulness of MCDA is needed | Cost-effectivness as criterion was included, but is redundant, other economic criteria are ‘impact on other spending’ and ‘budget impact on health plan’ |
| **Goetghebeur [9]** | MCDA estimate was 41% of maximum value. “Improvement of efficacy” (14% of total MCDA estimate), “Disease severity” (11%) and “Quality of evidence” (22%, for Q2 and Q3 combined) were the most impoartnat criteria. | Disucssions with participants highlighted the need of approbriate evidence, relevant outcome measures and the comparinson with alternatives. Some criteria were seen as redundant (“stakeholder pressure” (33%) and “clinical guidelines” (25%)) | Further research about the usefulness of MCDA is needed | Economic criterie were ‘cost-effectivness’, ‘impact on other spending’ and ‘budget impact on health plan’ |
| **Golan [10]** | The developed frameworks identiefed technologies that are good value for money. The best rated technologies were smoking cessation drugs and Taxotere. | Not beene applied in a real world setting yet | Intend to be applied in the Israeli Basket Committee | Economic criterion were total costs |
| **Hilgerink [11]** | The most important criterion is the diagnostic performace, particluary sensitivity. Overall, PAM is the prefered methods exept in the most negative scenario. | Engineers are more likely to put high weights on costs and effectivness than other groups. | In the further development of PAM, issues about early diagnosis should be examined more closely. | Costs were one of four criteria with the sub criteria: scan time, manpower, price and peripheral equitment, however the autors suggest to include costs only as overall criterion. |
| **Hummel [12]** | The strongest impact t if the new intervention is to reduce the costs of NFS and to optimize the prediction of progression. | None stated | The approach of combining AHP with a decision model is appropirabte for early healh technoogy assessment. | Costs was an main criterion splittet into investment in materials and treatment costs. |
| **Jehu-Appiah [13]** | Cost-effectivness and the targetd population group were the most important critreria. Interventions in child health, reproductive health and communicable disease received the highest ranking. | Only policy makers’ preferences, which may not necessarily reflect those of the general population | hearsay evidence shows that policymakers have used the present study findings as part of the development process of the third Five Year POW. | Preferences of costs and effectiveness were combined in one criteria |
| **Le Gales [14]** | A set of sreening stategies could be selected. These were different from strategies which were selected by pure cost-effectivness anaysis | Cosensus of experts give no guarentee that choices are rational from a societal viewpoint. Ethical issues include discrimination of certain ethinicities. | The results were proposed as clear reommendations for public health officials of the region. | Costs was one of seven criteria. |
| **Marsh [15]** | Taxation as prevntive strategy received the highest priority, followed by mass-media campaigns and brief interventions. | Ranking was mostly driven by the criterion cost-effectivness, qualitative criteria and local context haven’t been included. innovative, new, unknown interventions tend to be excluded. Decision-makers preferences can vary from preferences of the public. | None stated | 2 of 5 criteria: incremental cost-effectivness (cost per QALY), affordability (budget impact) |
| **Miot [16]** | 89% of participants thought 9 out of 14 of the MCDA criteria should always be considered. The criteria “Budget impact”, “Cost-effectiveness” and “Completeness of evidence” received the highest weights. | The Framework helped participants to understand the decision process. In the opinion of panellists the framework is easily adaptable to different health interventions. | The health plan considered to only fund LBC up to pap smears. After negotiating with the pathology laboratories, the fee for LBC was then reduced to be appropriate for full funding. | Considered Criteria: “Budget impact on health plan” , “Cost-effectiveness of intervention”, “Impact on other spending”, “Opportunity costs” |
| **Shin [17]** | Delivery of vacancies by private medical facilities obtained higher AHP values on all 3 levels of the hierarchy. | The vaccine adverse event reporting factor, networks, and partnerships between public health centres and private medical facilities factor were found to be the two most important criteria of all the factors | Not stated | Costs were included in several sub criteria like “Investment resources for infrastructure”, “governmental budget” and “economical satisfaction” |
| **Sloane [18]** | 1 of 3 alternatives had the highest overall score (0.825). Safety received the highest weight (0.317), followed by clinical factors (0.301), Biomedical Engineering (0.218), and Cost (0.163) | Costs had the at least weight (0.163) in the final model for several reasons like strategic importance of high-risk pregnancies and strong community funding of the hospital | AHP should be used as an integrative tool of comparing decision-making criteria | Costs had the at least weight (0.163) in the final model for several reasons like strategic importance of high-risk pregnancies and strong community funding of the hospital |
| **Tony [19]** | The intervention reached a MCDA estimate of 0.44, which was hard to interpret for participants. The most important criteria for participants were ‘improvement of efficiency’ and ‘relevance and validity of evidence’. ‘Disease | 6 non quantifiable contextual criteria (3 ethical, 3 other components) were included in the final decision. Utility had the highest positive impact on the score. In contrast, efficiency and opportunity costs had the lowest impact. | Further research about the usefulness of MCDA is needed, no explicit implementation of the final results. | Economic criteria were ‘cost-effectiveness’, ‘impact on other spending’ and ‘budget impact on health plan’ |
| **Venhorst [20]** | preliminary rating tool for assessing breast cancer interventions in LMICs consisting of 10 carefully crafted criteria | None stated | The developed tool can be enhanced to other diseases. | 2 economic criteria: cost-effectiveness and affordability |
| **Wilson [21]** | The screening programme was ranked first, prevention and acute service programmes followed because of the next best cost per point ratio. | Cost-effectiveness was determined by calculating the cost per point ratio. It would also be rational to include costs into the weighting process. | The tool is now in use across the PCT and will be evaluated and refined after its first year of operation. | Costs were included after weighting by calculating costs per point. |
| **Youngkong[22]** | Upon deliberation, policy makers expressed a preference for programs that target high risk groups. The community members preferred interventions that target the youth or the general population. Patient representatives gave all interventions the same priority. The rank order correlation between the priorities before and after deliberation was between 37% and 46%. | None stated | policy makers and community members , but not patient representatives agreed this MCDA approach approach | Effectiveness was already in DCE included, cost-effectiveness was added into the deliberative process |
| **Youngkong [23]** | 17 interventions were assessed, 9 were selected for further assessment, results were submitted to decision-makers | MCDA was adapted to the regional Thai context, Identification of true representatives of stakeholders was seen as difficult, unclear to what extend the final decision-making body made the decision up on the MCDA approach | The results of the MCDA study were presented to the NHSO’s Subcommittee for Development of Benefit package. This committee considered the results within their decision. | Effectiveness and economic impact of household expenditures in the first assessment. value for money (ICER), budget impact in the second assessment |

**Data extraction form 2: MCDA methodology**

| **First author** | **MCDA type** | **Selection of criteria** | **Number of criteria and criteria used** | **Weighting method** | **Obtaining the evidence** | **Scoring method** | **Practical issues, and limitations that may affect MCDA approach** |
| --- | --- | --- | --- | --- | --- | --- | --- |
| **Baeten [1]** | DCE | Criteria were obtained from another study [4] | 6 criteria: severity of disease, number of potential beneficiaries, age of target group, individual health benefits, poverty reduction, cost-effectiveness | Qualification (2-3 levels) of criteria was obtained from another study [2] | Existing breast cancer model from the WHO (CHOICE program), epidemiological data from the European region from Global Burden of disease Report and other literate sources | Interventions were ranked according to the sum of the weights of its criteria levels. The results were presented in a composite league table. | The preferences were obtained in other settings, therefore couldn’t be applicable, large amount of information is lost by putting it into different categories, limitations also occur by assessing interventions for the same disease |
| **Baltussen [3]** | DCE | Based on a previous survey in Uganda plus discussions with stakeholders and policy makers in Ghana | 6 criteria: cost-effectiveness, poverty reduction, age of target group, severity, health effects and total budget impact | All criteria on 2 levels, DCE was conducted with 30 health policy makers | Literature review about all criteria in WHO Report 2002, WHO Global Burden of disease, WHO CHOICE project, discussions with the authors and other sources | Interventions were ranked according to the sum of the weights of its criteria levels. The results were presented in a composite league table. | Preferences of costs and effectiveness were combined in one criteria, all attributes were only scaled in two levels, interactions between criteria could influence the results |
| **Baltussen [4]** | DCE | Two group discussions to identify the relevant criteria and related levels to be included in the DCE | 6 criteria: severity, number of beneficiaries, age, individual health benefit, poverty reduction, cost-effectiveness | 4 criteria on 2 levels and 2 criteria on 3 levels, DCE was conducted with 66 respondents (health professionals/ public health experts) | Literature review about criteria of health intervention in WHO databases (WHO-CHOICE, World Health Report 2002, etc.) and discussions with the authors | Composite league table where results of DCE and information about intervention are calculated to receive a final ranking of interventions | The views of high-level decision-makers were not included to the optimal extent, and led to the omission of criteria like budget-impact in the analysis. Therefore, the resulting priority list could change. |
| **Bots[5]** | SMART (simple attribute rating technique) | No statement about process | 2 main criteria: costs of care and health gain (with 4 sub criteria: life expectancy, quality of life, burden of treatment, prevalence) | Weights were determined by a group of key decision-makers in Dutch health policy: Changes in health status (63%) (Prevalence (28%), life expectancy (21%), quality of life (32%), burden of treatment (19%) and costs of care (37%) | Organizations (10%) and professionals (1%) in the Netherlands were contacted to find potential interventions for efficiency improvements. Experts filled impact matrix by ‘best guess’, scores with high disagreement were discussed in a consensus meeting. | Impact of options on criteria by scores on a 1-5 scale | This process consisted of 2 expert groups. The construction of the impact matrix was performed by medical experts, the weighting by policy-makers. This caused some disagreement. The description of issues could have a significant influence on the result. |
| **Cho [6]** | AHP | Basic principles such as ‘exclusiveness,’ ‘completeness’ and ‘optimum size’ for criteria | 3 criteria: marketability, technology applicability and public benefits, all with sub-criteria | Scale of 1 to 9 to pairwise compare alternatives and criteria on different levels | Expert opinions | Hierarchic composition is used to weight the eigenvectors in a level by the eigenvector weights of the corresponding criteria and the sum is then taken over all weighted eigenvector entries in the next lower level of the hierarchy. The resulting priorities are thus determined with respect to the overall goal of the hierarchy, which has the value 1. | Some participants preferred to make a simple evaluation through discussion. The AHP seemed complex to them. The high number of evaluated alternatives made selection difficult. |
| **Diaby [7]** | DCE | Asking the focus group | 4 criteria: cost-effectiveness, severity of the condition, socioeconomic group, and age group of patients | DCE (criterion “age group” was sorted out) | Cost effectiveness: Cost per QALY with the MUGEFCI as perspective with sensitivity analysis (4*4 matrix with 4 groups), Severity: WHO report “Global burden of disease” Social class: approximation from the MUGEFCI database | Cost effectiveness, Severity:” Social class: 2 groups | Simplified design: ‘costs’ and ‘effects’ categories into a single crude attribute, strongly influenced results by the choice of criteria for drug reimbursement, included attributes with a relatively low number of levels, adding more levels would have made DCE for respondents more difficult. |
| **Goetghebeur [8]** | Direct weighting, EVIDEM | EVIDEM framework | 15 criteria: Disease severity, Size of population affected by disease, Clinical guidelines, Comparative interventions limitations, Improvement of efficacy/effectiveness, Improvement of safety & tolerability, Improvement of patient reported outcomes, Public health interest, Type of medical service. Budget impact on health plan, Cost-effectiveness of intervention, Impact on other spending, Adherence to requirements of decision making body, Completeness and consistency of reporting evidence, Relevance and validity of evidence | weights to each MCDA decision criterion on a scale of 1 to 5 | Data for each criterion was identified by searching PubMed, websites of HTA agencies and the WHO. A by-criterion HTA report was synthesized following the EVIDEM methodology. Quality of evidence was assessed using a standardized approach including quality related criteria (Q1-3) | Scoring on a 4-point scale based on the HTA report | Participants had difficulties to define their perspective (individual vs. societal) . The number of technologies as well as the number of participants was limited. The methodology is still under development. |
| **Goetghebeur [9]** | Direct weighting, EVIDEM | EVIDEM framework | 14 criteria without “Adherence to requirements of decision making body” plus 6 non quantifiable contextual criteria ( 3 ethical: utility, efficiency, fairness, 3 other components: stakeholder pressure, System capacity and appropriate use of intervention, political context) | weights to each MCDA decision criterion on a scale of 1 to 5 | Synthesized HTA report was performed following the EVIDEM methodology. Quality of evidence was integrated into the MCDA process by the criteria Q1-3:“completeness and consistency of reporting” and “relevance and validity of evidence” | Scoring on a 4-point scale based on the HTA report plus evaluation of final results by extrinsic criteria | Some participants felt challenged by doing the complete MCDA within one panel session. The reassessment of criteria weights resulted in different weights which show that participants still have to get familiar with the MCDA approach and their perspective. |
| **Golan [10]** | PAPRIKA (Potentially All Pairwise RanKing of all possible Alternatives) | Literature research and observation of 11 countries | 4 criteria: benefit, including equity benefit, costs to health system, quality of evidence, additional ‘X-factors’ | Pairwise rank of hypothetical technologies | Data was obtained from realistic data used by the Basket Committee (various years) | Performance of technologies was determined by the first author | Rating exercises can be biased by uncertainty involved, the prioritization decision themselves can be different from the results of the framework. The ‘x-factor’ criterion can be critical for decision-making. |
| **Hilgerink [11]** | AHP | Criteria were obtained by literature research and expert interviews: | 4 criteria: costs, effectiveness, patient comfort, safety | Pairwise comparison of sub criteria and alternatives | Evidence from literature review, data of clinical prototypes and a comparative technology (PAM 2) was used | Individual judgements were aggregated to obtain the weighted impact on preferences | Cost should be used as a main criterion without sub criteria because of several overlaps. Only imaging techniques which are already in clinical use were compared against new intervention. |
| **Hummel [12]** | AHP | The expert panel selected criteria | 3 main criteria: health-related quality of life, complications during surgery, costs, spited into 7 sub criteria | Experts evaluated the relative importance of the criteria. Pairwise comparison of criteria and alternatives by using a nine-point scale | Literature research, the performance of the new technology was obtained by expert opinions. The data was used to generate a decision tree model. | The comparisons were used to calculate a group average. Weighting factors are calculated by the eigenvector approach. The sum of all weighted alternatives and criteria is the overall priority. | Expert judgements could replace clinical evidence, the selection of experts could have affected the results. |
| **Jehu- Appiah [13]** | DCE | discussion with 7 policy makers in Ghana | 5 criteria: cost-effectiveness, poverty reduction, age of target group, severity of disease, and number of potential beneficiaries | DCE survey consisted of 16 pairs of scenarios, 4 criteria were measured at 4 levels and one criteria at 2 levels | Literature review about all criteria in WHO Report 2002, WHO Global Burden of disease, WHO CHOICE project, discussions with the authors and other sources | Interventions were ranked according to the sum of the weights of its criteria levels. The results were presented in a composite league table. | DCE could only include criteria with max 3 levels, right implementation of economic aspects not clear |
| **Le Gale [14]** | Direct weighing and outranking (ELECTRE 1S Model) | Debates inside the study group | 7 criteria: effectiveness, total costs, technical feasibility, practical feasibility, ethical acceptability, information follow-up in time, impact on heath education | 5 criteria were direct weighted on a 5-point scale, 2 criteria (costs and effectiveness) directly included into the outranking method | Expert opinions on 5 direct weighted criteria, data about effectiveness and costs were obtained from regional laboratories and local officials | Using a MCDA outranking model (ELECTRE 1S), all criteria were assessed | No limitations stated |
| **Marsh [15]** | DCE | Literature review about MCDA and workshop with decision-makers | 5 criteria (Incremental cost-effectiveness (cost per QALY), eligible population, distribution of benefits, affordability (budget impact), certainty), | DCE with 16 choice sets | Literature review, decision modelling, institutional data | A utility model included the evidence measures to calculate final utility of an intervention | Exclusion of 2 criteria: feasibility and acceptability due to qualitative characteristics, more qualitative approach would be more appropriate to match the local decision context. |
| **Miot [16]** | Direct weighting, EVIDEM | EVIDEM framework, 14 of 15 criteria without “Adherence to requirements of decision-making body” plus 4 of 6 contextual criteria | 14 criteria without “Adherence to requirements of decision making body” plus 4 qualitative criteria (Impact on future decisions, relationship with pathology providers, Impact on screening intervals, Patient expectation) | weights to each MCDA decision criterion on a scale of 1 to 5 | Data for each criterion was identified by searching PubMed, websites of HTA agencies and the study related health plan. A by-criterion HTA report was produced. Quality of evidence was assessed using a standardized approach including quality related criteria (Q1-3) | Scoring on a 4-point scale based on the HTA report | No limitation stated |
| **Shin [17]** | AHP | Delphi method with health care professionals | 3 main criteria: program infrastructure, program process, program outcome and 22 sub criteria on 3 levels | Pairwise comparison with numerical scale from 1 to 9 | Literature review, expert opinions | The overall priority is calculated by multiplying the local priorities with their corresponding weights along the hierarchy. Finally, a preference gap between alternatives was calculated. | No limitations stated |
| **Sloane [18]** | AHP | Discussions with participants/ experts in the field | 4 main criteria: safety, clinical factors, biomedical engineering, cost | Iterative, pairwise comparison with numerical scale from 1 to 9 | Expert opinion, data from manufactures and hospital | The overall priority is calculated by multiplying the local priorities with their corresponding weights along the hierarchy. Finally, a preference gap between alternatives was calculated. | None stated |
| **Tony [19]** | Direct weighting, EVIDEM | EVIDEM framework | 14 criteria without “Adherence to requirements of decision making body” plus 6 non quantifiable contextual criteria ( 3 ethical: utility, efficiency, fairness, 3 other components: stakeholder pressure, System capacity and appropriate use of intervention, political context) | weights to each MCDA decision criterion on a scale of 1 to 5 | An extensive analysis of the published and grey literature was performed to identify relevant data. Synthesized HTA report on was performed following the EVIDEM methodology. Quality of evidence was assessed using a standardized approach including quality related criteria (Q1-3) | Scoring on a 4-point scale based on the HTA report | The MCDA estimate of 0.44 was hard to interpret for participants as a single value. The reassessment of criteria weights resulted in different weights which show that participants still have to get familiar with the MCDA approach and the perspective. |
| **Venhorst [20]** | Direct weighting) | Literature review about decision criteria and delphi panel with experts | Effectiveness, quality of the evidence, magnitude of individual health impact, acceptability, cost-effectiveness, technical complexity, affordability, safety, geographical coverage, and accessibility | weights to each MCDA decision criterion on a scale of 1 to 5 | Not stated | Scores to each MCDA decision criterion on a scale of 0 to 2 | Time consuming workload for experts, definition of scoring scale, criteria overlaps, missing evidence, no input form patients and local health worker |
| **Wilson [21]** | Weighted benefit score (WBS) | Brainstorming session, mutually exclusive and exhausting choice | 7 criteria: access and equity, effectiveness, priorities, need, prevention, process, quality of life | Allocation of 100 percentage points among 7 criteria | Not stated (assessment of fictitious proposals) | Scoring on a 10-point scale (5 means no change), afterwards multiplication of scores and weights. The finals scores were combined with cost of options, which resulted in a cost/score ratio. | Costs for a point of benefit are the same among different intervals and programmes. Criteria could be not exhausting. |
| **Youngkong [22]** | DCE with deliberation process | DCE, group discusssions with policy macers, patients and health volunteers: | 6 criteria: target groups of interventions, gender of target, groups, type of interventions, effectiveness, quality of evidence | DCE, construction of a performance matrix, using a logistic regression model to estimate the selection probability | An international systematic review on HIV prevention strategies The main outcomes were changes in HIV risk behaviour, HIV incidence, cost per HIV infection averted or cost per quality-adjusted life year (QALY) gained. All studies were assessed b certain quality criteria. [24] | using a logistic regression model to estimate the selection probability, afterwards delibartive process with stakeholders to reassess ranking order | Cognitive demanding DCE may not be appropriate for all stakeholders. The intervention set was homogeneous in terms of the criteria which resulted in low variation in the probabilities of inclusion. Not all stakeholders were engaged in a single deliberative process to arrive at a consensus on the rank ordering. |
| **Youngkong [23]** | Direct weighting (with consideration of DCE study) | Identification of criteria by literature rview, consultation with two working groups and considaration of a DCE study to match the Thai context | 6 criteria: Size of population, sverity of disease, effectivness, variation in practisce, economic impact on household expenditure, euity/ethical and social implication plus 2 criteria: value for money (ICER), budget impact | For 6 criteria: equal weights, for 2 criteria: consideration of numerical measures | Not stated | For six criteria: scale of 1-5, for 2 criteria: consideration of numerical measures | Definition of scoring scales difficult, incomplete evidence, some criteria difficult to understand for non-academics, weights were equal for all criteria |

1. Baeten SA, Baltussen RM, Uyl-de Groot CA, Bridges J, Niessen LW: **Incorporating equity-efficiency interactions in cost-effectiveness analysis-three approaches applied to breast cancer control**. *Value in health : the journal of the International Society for Pharmacoeconomics and Outcomes Research* 2010, **13**(5):573-579.

2. Baltussen R, Youngkong S, Paolucci F, Niessen L: **Multi-criteria decision analysis to prioritize health interventions: Capitalizing on first experiences**. *Health policy (Amsterdam, Netherlands)* 2010, **96**(3):262-264.

3. Baltussen R, Stolk E, Chisholm D, Aikins M: **Towards a multi-criteria approach for priority setting: an application to Ghana**. *Health economics* 2006, **15**(7):689-696.

4. Baltussen R, ten Asbroek AH, Koolman X, Shrestha N, Bhattarai P, Niessen LW: **Priority setting using multiple criteria: should a lung health programme be implemented in Nepal?** *Health policy and planning* 2007, **22**(3):178-185.

5. Bots PWG, Hulshof JAM: **Designing multi-criteria decision analysis processes for priority setting in health policy**. *Journal of Multi-Criteria Decision Analysis* 2000, **9**(1-3):56-75.

6. Cho KT, Kim SM: **Selecting medical devices and materials for development in Korea: the analytic hierarchy process approach**. *The International journal of health planning and management* 2003, **18**(2):161-174.

7. Diaby V, Lachaine J: **An application of a proposed framework for formulary listing in low-income countries: the case of Cote d'Ivoire**. *Applied health economics and health policy* 2011, **9**(6):389-402.

8. Goetghebeur MM, Wagner M, Khoury H, Levitt RJ, Erickson LJ, Rindress D: **Bridging health technology assessment (HTA) and efficient health care decision making with multicriteria decision analysis (MCDA): Applying the evidem framework to medicines appraisal**. *Medical Decision Making* 2012, **32**(2):376-388.

9. Goetghebeur MM, Wagner M, Khoury H, Rindress D, Gregoire JP, Deal C: **Combining multicriteria decision analysis, ethics and health technology assessment: applying the EVIDEM decision-making framework to growth hormone for Turner syndrome patients**. *Cost effectiveness and resource allocation : C/E* 2010, **8**:4.

10. Golan O, Hansen P: **Which health technologies should be funded? A prioritization framework based explicitly on value for money**. *Israel journal of health policy research* 2012, **1**(1):44.

11. Hilgerink MP, Hummel MJ, Manohar S, Vaartjes SR, Ijzerman MJ: **Assessment of the added value of the Twente Photoacoustic Mammoscope in breast cancer diagnosis**. *Medical devices (Auckland, NZ)* 2011, **4**:107-115.

12. Hummel JM, Boomkamp IS, Steuten LM, Verkerke BG, Ijzerman MJ: **Predicting the health economic performance of new non-fusion surgery in adolescent idiopathic scoliosis**. *Journal of orthopaedic research : official publication of the Orthopaedic Research Society* 2012, **30**(9):1453-1458.

13. Jehu-Appiah C, Baltussen R, Acquah C, Aikins M, d'Almeida SA, Bosu WK, Koolman X, Lauer J, Osei D, Adjei S: **Balancing equity and efficiency in health priorities in Ghana: the use of multicriteria decision analysis**. *Value in health : the journal of the International Society for Pharmacoeconomics and Outcomes Research* 2008, **11**(7):1081-1087.

14. Le Gales C, Moatti JP: **Searching for consensus through multi-criteria decision analysis. Assessment of screening strategies for hemoglobinopathies in southeastern France**. *International journal of technology assessment in health care* 1990, **6**(3):430-449.

15. Marsh K, Dolan P, Kempster J, Lugon M: **Prioritizing investments in public health: a multi-criteria decision analysis**. *Journal of public health (Oxford, England)* 2012.

16. Miot J, Wagner M, Khoury H, Rindress D, Goetghebeur MM: **Field testing of a multicriteria decision analysis (MCDA) framework for coverage of a screening test for cervical cancer in South Africa**. *Cost effectiveness and resource allocation : C/E* 2012, **10**(1):2.

17. Shin T, Kim CB, Ahn YH, Kim HY, Cha BH, Uh Y, Lee JH, Hyun SJ, Lee DH, Go UY: **The comparative evaluation of expanded national immunization policies in Korea using an analytic hierarchy process**. *Vaccine* 2009, **27**(5):792-802.

18. Sloane EB, Liberatore MJ, Nydick RL, Luo W, Chung QB: **Using the analytic hierarchy process as a clinical engineering tool to facilitate an iterative, multidisciplinary, microeconomic health technology assessment**. *Computers &amp; Operations Research* 2003, **30**(10):1447-1465.

19. Tony M, Wagner M, Khoury H, Rindress D, Papastavros T, Oh P, Goetghebeur MM: **Bridging health technology assessment (HTA) with multicriteria decision analyses (MCDA): Field testing of the EVIDEM framework for coverage decisions by a public payer in Canada**. *BMC health services research* 2011, **11**.

20. Venhorst K, Zelle S, Tromp N, Lauer J: **Multi-criteria decision analysis of breast cancer control in low- and middle- income countries: development of a rating tool for policy makers**. *Cost Effectiveness and Resource Allocation* 2014, **12**(1):13.

21. Wilson E, Rees J, Fordham R: **Developing a prioritisation framework in an English Primary Care Trust**. *Cost Effectiveness and Resource Allocation* 2006, **4**(1):3.

22. Youngkong S, Teerawattananon Y, Tantivess S, Baltussen R: **Multi-criteria decision analysis for setting priorities on HIV/AIDS interventions in Thailand**. *Health Research Policy and Systems* 2012, **10**(1):1-8.

23. Youngkong S, Baltussen R, Tantivess S, Mohara A, Teerawattananon Y: **Multicriteria decision analysis for including health interventions in the universal health coverage benefit package in Thailand**. *Value in health : the journal of the International Society for Pharmacoeconomics and Outcomes Research* 2012, **15**(6):961-970.

24. Pattanaphesaj J, Teerawattananon Y: **Reviewing the evidence on effectiveness and cost-effectiveness of HIV prevention strategies in Thailand**. *BMC Public Health* 2010, **10**:401.
